# The effect of size, charge, and peptide ligand length on kidney targeting by small, organic nanoparticles

**DOI:** 10.1002/btm2.10173

**Published:** 2020-07-30

**Authors:** Yi Huang, Kairui Jiang, Xuting Zhang, Eun Ji Chung

**Affiliations:** ^1^ Department of Biomedical Engineering University of Southern California Los Angeles California USA; ^2^ Department of Chemical Engineering and Materials Science University of Southern California Los Angeles California USA; ^3^ Department of Medicine, Division of Nephrology and Hypertension University of Southern California Los Angeles California USA; ^4^ Department of Surgery, Division of Vascular Surgery and Endovascular Therapy University of Southern California Los Angeles California USA

**Keywords:** chronic kidney disease, micelle, nanoparticle, peptide, renal clearance

## Abstract

Chronic kidney disease (CKD) affects 15% of the US adult population. However, most clinically available drugs for CKD show low bioavailability to the kidneys and non‐specific uptake by other organs which results in adverse side effects. Hence, a targeted, drug delivery strategy to enhance kidney drug delivery is highly desired. Recently, our group developed small, organic nanoparticles called peptide amphiphile micelles (PAM) functionalized with the zwitterionic peptide ligand, (KKEEE)_3_K, that passage through the glomerular filtration barrier for kidney accumulation. Despite high bioavailability to the kidneys, these micelles also accumulated in the liver to a similar extent. To further optimize the physicochemical properties and develop design rules for kidney‐targeting micelles, we synthesized a library of PAMs of varying size, charge, and peptide repeats. Specifically, variations of the original (KKEEE)_3_K peptide including (KKEEE)_2_K, (KKEEE)K, (EEKKK)_3_E, (EEKKK)_2_E, (EEKKK)E, KKKKK, and EEEEE were functionalized onto nanoparticles, and peptide surface density and PEG linker molecular weight were altered. After characterization with transmission electron microscopy (TEM) and dynamic light scattering (DLS), nanoparticles were intravenously administered into wildtype mice, and biodistribution was assessed through ex vivo imaging. All micelles localized to the kidneys, but nanoparticles that are positively‐charged, close to the renal filtration size cut‐off, and consisted of additional zwitterionic peptide sequences generally showed higher renal accumulation. Upon immunohistochemistry, micelles were confirmed to bind to the multiligand receptor, megalin, and histological analyses showed no tissue damage. Our study provides insight into the design of micelle carriers for kidney targeting and their potential for future therapeutic application.

## 1 INTRODUCTION

Chronic kidney disease (CKD) affects 600 million people globally and is characterized by gradual kidney function decline.^1^ A common form of CKD is autosomal dominant polycystic kidney disease (ADPKD), which affects over 12 million people worldwide and is characterized by renal cyst formation that can result in the need for dialysis or kidney transplant.^2^, ^3^, ^4^, ^5^ Currently, there is no cure for ADPKD and tolvaptan is the only FDA approved treatment for ADPKD.^6^ Despite enthusiasm, tolvaptan has been reported to cause several side effects including polyuria, thirst, nocturia, and drug‐induced liver damage.^7^, ^8^, ^9^, ^10^ According to a study of 961 ADPKD patients undergoing tolvaptan treatment, the adverse event rates for thirst and nocturia were found to be approximately 55% and 29%.^9^ Polyuria affected 38% of patients and was the primary reason for dose reduction and drug discontinuation due to its significant impact on patients' quality of life.^11^, ^12^ Moreover, tolvaptan is known to cause liver toxicity and elevated liver enzyme levels, which may further deteriorate liver health, as liver cysts and fibrosis are common extrarenal manifestations of ADPKD.^8^, ^13^ As a result, frequent monitoring of liver function is required for patients on tolvaptan treatment.^14^, ^15^ Like tolvaptan, many drugs for CKD lead to non‐specific uptake and undesired adverse effects,^16^, ^17^ and hence, the development of a targeted drug delivery platform that can directly unload drugs to diseased kidneys has the potential to reduce systemic side effects without compromising therapeutic efficacy.

To that end, our group has recently developed small, organic nanoparticles called peptide amphiphile micelles (PAMs, ~15 nm in diameter) to target the kidneys by incorporating the zwitterionic peptide ligand, (KKEEE)_3_K([Lys–Lys–Glu–Glu–Glu]_3_–Lys), which has been found to bind to megalin, a multiligand receptor highly expressed on renal proximal tubule cells.^18^, ^19^, ^20^ Upon intravenous injection of (KKEEE)_3_K PAMs into wildtype mice, although (KKEEE)_3_K PAMs were found to accumulate in the kidneys to a high degree, they also accumulated similarly in the liver, likely due to the mononuclear phagocytic system (MPS).^19^

**FIGURE 1:**
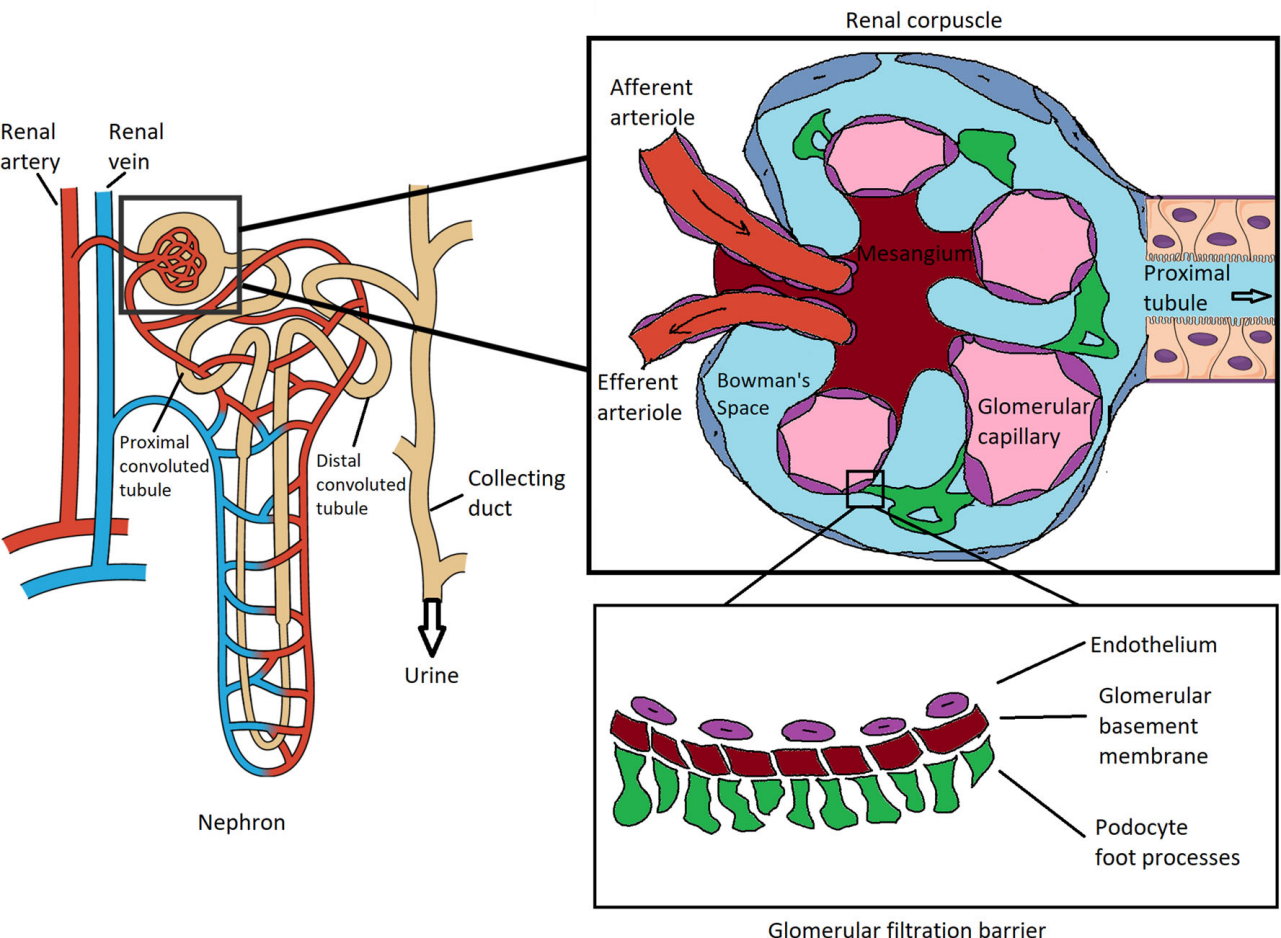
Schematic of the nephron, renal corpuscle, and the glomerular filtration barrier

The kidneys filter blood and each kidney is composed of approximately a million functional units called nephrons. Each nephron consists of two main parts, the renal tubule and the renal corpuscle which contains a network of blood capillaries called the glomerulus. When blood enters the glomerulus, a fraction of blood plasma will pass through the glomerular filtration barrier (GFB) and into the renal tubule system to be processed into urine (Figure 1).^21^ Specifically, the GFB is comprised of three components and includes the endothelium, glomerular basement membrane (GBM), and podocyte foot processes that collectively acts as a size‐selective and charge‐selective sieve during the filtration of macromolecules.^21^, ^22^, ^23^ The pores present in each of these layers include fenestrations of the endothelium (70–90 nm), a mesh created through a network of collagen, laminin, and proteoglycans that make up the GBM (~8 nm), and filtration slits of the podocyte foot processes (4–11 nm).^21^, ^24^ Regarding charge, both the endothelium and podocytes express glycocalyx on their membranes, mostly in the form of heparan sulfate (over 50%), which is negatively‐charged.^25^, ^26^, ^27^ The GBM is also negatively‐charged due to the presence of anionic proteoglycans (e.g., heparan‐sulfate and chondroitin‐sulfate glycosaminoglycan), and hence, positively‐charged nanoparticles have been reported to passage through the GFB more readily due to charge repulsion upon interaction with negatively‐charged macromolecules.^28^, ^29^

While most nanoparticles are too large to cross the fenestration of the GFB and will be recognized by the MPS system, ultrasmall nanoparticles (<8 nm) have been reported to pass through the GFB and clear via renal clearance.^30^ Since the majority of cyst development in CKD such as PKD occurs in the tubules,^31^, ^32^ one strategy for targeted drug delivery for PKD is to design nanoparticles that can pass through the GFB through their physicochemical properties, but be retained in proximal tubule through the addition of targeting ligands. Hence, to study how size, charge, peptide ligand length, and surface ligand density affects nanoparticle kidney‐targeting ability and to optimize the kidney‐targeting potential of PAMs, we developed a library of micelles by incorporating variations of the original zwitterionic (KKEEE)_3_K peptide including (KKEEE)_2_K, (KKEEE)K, (EEKKK)_3_E, (EEKKK)_2_E, (EEKKK)E, KKKKK, and EEEEE. The size and surface charge of the micelle library was characterized by TEM, DLS, and zeta potential, and to assess nanoparticle kidney‐targeting ability, micelles were intravenously administered into C57B/6J mice. Kidney targeting and micelle biodistribution was evaluated via ex vivo imaging after 24 hours. Moreover, micelle targeting to megalin was evaluated through immunohistochemistry, and kidney biocompatibility was assessed through histology, blood urea nitrogen, and urine creatinine.

## 2 RESULTS AND DISCUSSION

### 2.1 Synthesis and characterization of PAMs

The kidney‐targeting peptide (KKEEE)_3_K was found to accumulate in the kidneys in part by binding to the megalin receptor expressed on proximal tubule cells and is composed of three repeats of the peptide sequence, KKEEE.^19^ Previously, when (KKEEE)_3_K was incorporated into micelles and assessed in vivo, their biodistribution profile showed enhanced kidney accumulation but also liver accumulation of 35% likely due to the MPS system as (KKEEE)_3_K PAMs were 15 nm in diameter and larger than the cut off reported for passage through the GFB (8–10 nm).^19^ To optimize the physicochemical properties of PAMs, herein, we synthesized micelles with fewer repeats of KKEEE to test how peptide repeat number affected kidney accumulation. Moreover, since micelles are self‐assembled from monomers containing DSPE hydrophobic tails linked to polyethylene glycol (PEG), PEG molecular weight was also varied to further study the size effects on the renal targeting ability of nanoparticles. In addition to peptide repeat number and size, peptide sequences of opposite charge (e.g., EEKKK) as well as non‐zwitterionic peptide sequences (e.g., KKKKK and EEEEE) were incorporated to test the charge selectivity of the GFB on micelles. Furthermore, to assess how peptide ligand surface density affects renal targeting ability, we synthesized micelles with 50% or 100% peptide surface presentation.

**FIGURE 2:**
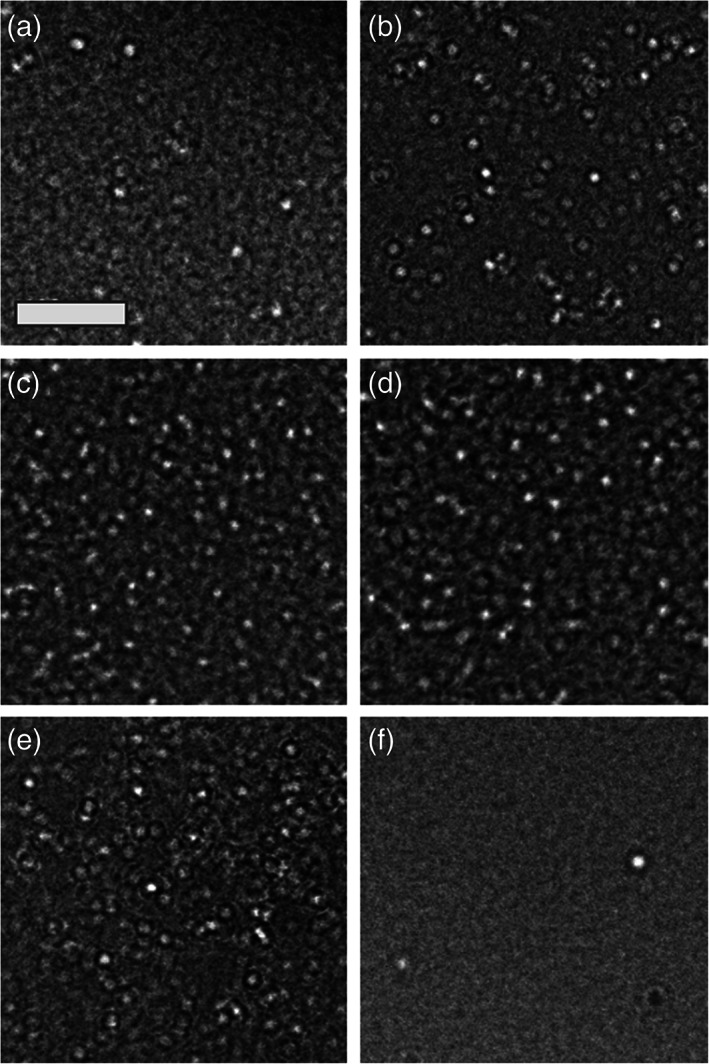
TEM images of micelles. (a) 50% PEG2000‐(KKEEE)_3_K, (b) 50% PEG2000‐(EEKKK)_3_E, (c) 50% PEG2000‐KKKKK, (d) 50% PEG2000‐EEEEE, (e) 50% PEG1000‐(KKEEE)_3_K, and (f) 100% PEG5000‐(KKEEE)_3_K show spherical morphology. Scale bar: 100 nm

To characterize the micelles, PAMs were self‐assembled in water or PBS at 100 μM (which is above the critical micelle concentration of ~1 μM^33^) and assessed via TEM and DLS. TEM images demonstrated spherical morphology of all PAMs (Figure 2), and DLS confirmed that decreasing the number of KKEEE repeats on nanoparticles correlated with a slight decrease in hydrodynamic diameter, although the diameter of all micelles were close in range to one another: PEG2000‐(KKEEE)_3_K (16.6 ± 2.5 nm), PEG2000‐(KKEEE)_2_K (12.2 ± 0.6 nm), PEG2000‐(KKEEE)K (10.6 ± 1.7 nm); PEG2000‐(EEKKK)_3_E (12.4 ± 1.2 nm), PEG2000‐(EEKKK)_2_E (14.0 ± 1.8 nm), PEG2000‐(EEKKK)E (11.8 ± 1.5 nm). PEG2000‐KKKKK and PEG2000‐EEEEE had an average diameter of 11.2 ± 1.2 nm and 10.2 ± 1.3 nm, which were similar to PEG2000‐(KKEEE)K and PEG2000‐(EEKKK)E due to the shorter peptide sequence (Table 1, Figure S1). As expected,^34^ increasing the PEG molecular weight to 5,000 resulted in a slight increase in diameter of nanoparticles to 17.2 ± 1.8 nm, and nanoparticles consisting of DSPE‐PEG1000 had a diameter of 10.4 ± 1.8 nm (Table 1).

**TABLE 1:** Characterization of PAMs by DLS and zeta potential

NP	Peptide surface density (%)	Diameter (nm)	Zeta potential (mV)	Net charge
PEG2000‐(KKEEE)_3_K	50	16.6 ± 2.5	−0.6 ± 1.4	−2
PEG2000‐(KKEEE)_2_K	50	12.2 ± 0.6	−0.2 ± 0.3	−1
PEG2000‐(KKEEE)K	50	10.6 ± 1.7	−1.1 ± 0.6	0
PEG2000‐(EEKKK)_3_E	50	12.4 ± 1.2	0.03 ± 0.1	+2
PEG2000‐(EEKKK)_2_E	50	14.0 ± 1.8	−3.1 ± 6.4	+1
PEG2000‐(EEKKK)E	50	11.8 ± 1.5	−0.1 ± 0.7	0
PEG2000‐KKKKK	50	11.2 ± 1.2	0.1 ± 0.2	+5
PEG2000‐EEEEE	50	10.2 ± 1.3	−40.9 ± 4.6	−5
PEG2000‐(KKEEE)_3_K	100	10.6 ± 0.4	−41.4 ± 2.9	−2
PEG2000‐(EEKKK)_3_E	100	15.0 ± 0.4	14.3 ± 1.6	+2
PEG**1000**‐(KKEEE)_3_K	50	10.4 ± 1.8	−2.9 ± 2.9	−2
PEG**5000**‐(KKEEE)_3_K	100	17.2 ± 1.8	−9.6 ± 1.0	−2
PEG2000‐NT	100	7.5 ± 0.1	−3.2 ± 0.8	0

Interestingly, despite (KKEEE)_3_K and (EEKKK)_3_E having an opposite net charge of −2 and + 2 derived from additional negatively‐charged glutamic acid (E) or positively‐charged lysine (K), the zeta potential of both micelles were found to be near neutral at −0.6 ± 1.4 mV and 0.03 ± 0.1 mV, respectively (Table 1). Similarly, the zeta potentials of PEG2000‐(KKEEE)_2_K (net −1), PEG2000‐(EEKKK)_2_E (net +1), PEG2000‐(KKEEE)K (net 0), and PEG2000‐(EEKKK)E (net 0) were also found to be near neutral at −0.2 ± 0.3 mV, −3.1 ± 6.4 mV, −1.1 ± 0.6 mV, and − 0.1 ± 0.7 mV. Since all these micelles consisted of 50:50 molar ratio of peptide‐DSPE‐PEG2000 to DSPE‐PEG2000‐methoxy, it is possible that the near‐neutral charge of DSPE‐PEG2000‐methoxy amphiphiles masked any charge differences of the micelles resulting in neutral zeta potentials. As the zeta potential of DSPE‐PEG2000‐NT (consisting of 100% DSPE‐PEG2000‐methoxy) was found to be near neutral (−3.2 ± 5.4 mV), the addition of 50% molar ratio of DSPE‐PEG2000‐methoxy may have affected their surface charge, leading to the resultant near‐neutral zeta potential measurements of PEG2000‐(KKEEE)_3_K and PEG2000‐(EEKKK)_3_E. This was also the case with nanoparticles consisting of 50% KKKKK, which had a zeta potential of 0.07 ± 0.2 mV (Table 1). On the other end, when the peptide sequence was altered to 50% EEEEE, micelles consisted of a negative zeta potential of −40.9 ± 4.6 mV.

Given these results, we synthesized micelles consisting of entirely PEG2000‐(KKEEE)_3_K and PEG2000‐(EEKKK)_3_ *E*. Zeta potential measurements of 100% PEG2000‐(KKEEE)_3_K and 100% PEG2000‐(EEKKK)_3_E micelles were found to be −41.4 ± 2.9 mV and 14.3 ± 1.6 mV, which is more consistent with the net charge of peptide sequence, −2 and + 2, respectively (Table 1).

### 2.2 In vivo renal targeting and biodistribution

To test the effects of nanoparticle characteristics on renal targeting, Cy7‐labeled micelles were intravenously administered into 6–7 week old male and female C57B/6 J mice and after 24 hours, micelle accumulation was assessed via ex vivo imaging of the kidneys, brain, lung, heart, liver, spleen, intestine, and bladder. 10 mol% of Cy7 was included into micelles to maximize the fluorescence signal without quenching and micelles with 45:45:10 molar ratio of DSPE‐PEG‐methoxy:DSPE‐PEG‐peptide:DSPE‐PEG‐Cy7 or 90:10 molar ratio of DSPE‐PEG‐peptide:DSPE‐PEG‐Cy7 were synthesized for in vivo studies.^35^ As shown in Figure S2, despite all micelles being at or above the reported renal filtration cut‐off size, all micelles accumulated in the kidneys to a greater extent than all other organs with the exception of 90% PEG5000‐(KKEEE)_3_K (Figure S2).^19^ 90% PEG5000‐(KKEEE)_3_K, which had the highest PEG molecular weight and largest diameter of 17.2 ± 1.8 nm (Table 1), mostly accumulated in the liver (1.6 × 10^9^ ± 1.3 × 10^8^ p/s/cm^2^/sr) likely via recognition of the MPS system (Figure S3). However, this was not statistically significant with its accumulation in the kidneys, which indicates an ability to simultaneously pass through the GFB and accumulate in the kidneys (Figures S2 and S3). Although the size of micelles was slightly larger than the cut‐off diameter of glomerular filtration (~10 nm), kidney accumulation and passage through the GFB has been found for 60–100 nm polycation‐siRNA nanoparticles and other soft macromolecules with the diameter beyond the cut‐off size of 8–10 nm.^24^, ^36^

#### 2.2.1 Biodistribution of PAMs with varying number of peptide repeats

**FIGURE 3:**
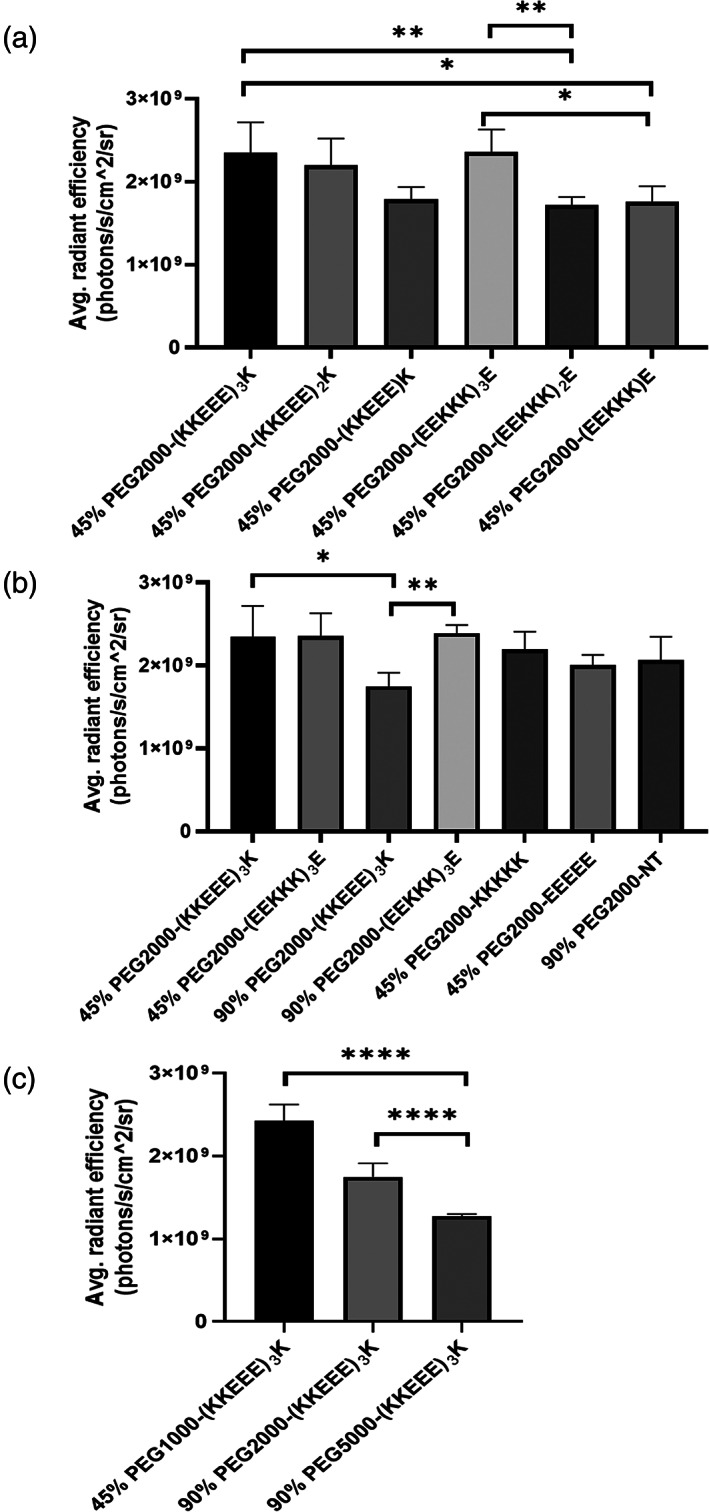
Kidney accumulation of micelles upon intravenous administration after 24 hours. Comparison of micelles containing (a) varying number of peptide repeats of KKEEE or EEKKK, (b) charge differences, and (c) (KKEEE)_3_K with various PEG molecular weight. *n* = 4. *p < .05, **p < .01, ****p < .0001

In Figure 3a, the effect of KKEEE/EEKKK repeats on nanoparticle kidney targeting in vivo was evaluated. Although we hypothesized that peptides with fewer repeats of KKEEE/EEKKK would decrease the overall molecular weight and size of micelles and thereby improve the ability of nanoparticles to pass through the GFB and accumulate in the kidneys, as shown in Figure 3a, additional sequence repeats trended towards having higher renal accumulation. Specifically, for micelles with 45% peptide surface presentation, PEG2000‐(KKEEE)_3_K (2.3 × 10^9^ ± 3.7 × 10^8^ p/s/cm^2^/sr) had the highest fluorescence intensity, followed by PEG2000‐(KKEEE)_2_K (2.2 × 10^9^ ± 3.3 × 10^8^ p/s/cm^2^/sr) and PEG2000‐(KKEEE)K (1.8 × 10^9^ ± 1.5 × 10^8^ p/s/cm^2^/sr, Figure 3a, although not statistically significant). Similarly, for PAMs with EEKKK repeats, PEG2000‐(EEKKK)_3_E (2.4 × 10^9^ ± 2.7 × 10^8^ p/s/cm^2^/sr) showed higher renal fluorescence intensity than PEG2000‐(EEKKK)_2_E (1.7 × 10^9^ ± 1.0 × 10^8^ p/s/cm^2^/sr, *p* < .01). PEG2000‐(EEKKK)E had a fluorescence intensity of 1.8 × 10^9^ ± 1.9 × 10^8^ p/s/cm^2^/sr, which was also statistically lower compared to PEG2000‐(EEKKK)_3_E (*p* < .05, Figure 3a). Given that KKEEE and EEKKK are zwitterionic peptides, one possible explanation for the higher kidney accumulation of micelles consisting of PEG2000‐(KKEEE)_3_K and PEG2000‐(EEKKK)_3_E micelles is that the additional peptide repeats provided enhanced zwitterionic characteristics and hence, resistance to nonspecific protein and opsonin absorption as well as liver uptake via the MPS system.^37^, ^38^, ^39^, ^40^, ^41^, ^42^ Additionally, given that the hydrodynamic diameter of the micelles with varying peptide repeats fell within a narrow range (Table 1 and Figure S1), peptide sequence and ligand length may play a more important role in the renal accumulation of micelles rather than size, which will be further probed in future studies.

#### 2.2.2 Biodistribution of PAMs with opposite charge

In Figure 3b, we compared the charge effects on micelle renal targeting. Despite having an opposite net charge of −2 and + 2, PEG2000‐(KKEEE)_3_K and PEG2000‐(EEKKK)_3_E with 45% surface peptide density accumulated in the kidneys to a similar extent (2.3 × 10^9^ ± 3.7 × 10^8^ p/s/cm^2^/sr and 2.4 × 10^9^ ± 2.7 × 10^8^ p/s/cm^2^/sr, respectively, Figure 3b). As found in Table 1, the zeta potentials of both micelles were near neutral due to the incorporation of PEG2000‐methoxy, which likely masked any charge differences that contributed to kidney targeting. Moreover, as mentioned, both peptides are zwitterionic and nanoparticles coated with zwitterionic materials have been reported to resist serum protein adsorption in vivo due to the highly hydrophilic surface and anti‐fouling properties.^43^, ^44^ On the other hand, micelles consisting of 90% PEG2000‐(KKEEE)_3_K, which had zeta potential of −41.4 ± 2.9 mV, had lower kidney accumulation (1.8 × 10^9^ ± 1.7 × 10^8^ p/s/cm^2^/sr) than the positively‐charged (14.3 ± 1.6 mV) 90% PEG2000‐(EEKKK)_3_E (2.4 × 10^9^ ± 1.0 × 10^8^ p/s/cm^2^/sr, *p* < .05). These results correlated with findings by Liang et al and Balogh et al that reported the GFB is a charge‐selective barrier in which positively‐charged nanoparticles pass through the GFB more easily than negatively‐charged nanoparticles and macromolecules due to electrostatic repulsion of the GBM.^45^, ^46^, ^47^, ^48^ In addition, 90% PEG2000‐(KKEEE)_3_K had significant higher liver uptake (1.6 × 10^9^ ± 5.0 × 10^7^ p/s/cm^2^/sr) than 90% PEG2000‐(EEKKK)_3_E (1.0 × 10^9^ ± 9.9 × 10^7^ p/s/cm^2^/sr, *p* < .0001, Figure S4). The higher liver uptake of negatively‐charged 90% PEG2000‐(KKEEE)_3_K was similar to the results by Xiao et al that reported negatively‐charged PEG‐oligocholic acid based micellar nanoparticles (−26.9 ± 1.7 mV) have higher liver uptake than positively‐charged counterparts (3.6 ± 0.8 mV) that were similar in size (18 ~ 21 nm).^49^

#### 2.2.3 The effects of PEG molecular weight and size on the biodistribution of micelles

**FIGURE 4:**
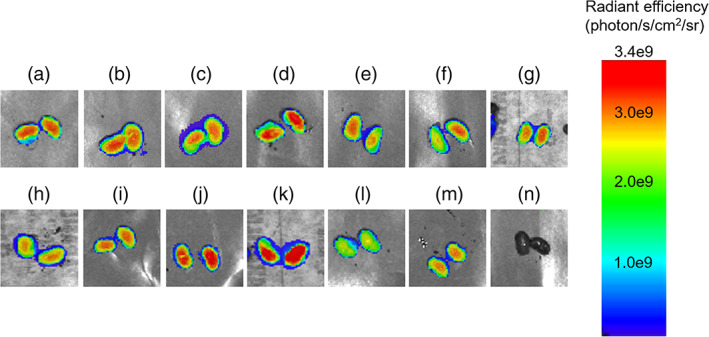
Ex vivo images of kidney fluorescence after (a) 45% PEG2000‐(KKEEE)_3_K, (b) 45% PEG2000‐(KKEEE)_2_K, (c) 45% PEG2000‐(KKEEE)K, (d) 45% PEG2000‐(EEKKK)_3_E, (e) 45% PEG2000‐(EEKKK)_2_E, (f) 45% PEG2000‐(EEKKK)E, (g) 45% PEG2000‐KKKKK, (h) 45% PEG2000‐EEEEE, (i) 90% PEG2000‐(KKEEE)_3_K, (j) 90% PEG2000‐(EEKKK)_3_E, (k) 45% PEG1000‐(KKEEE)_3_K, (l) 90% PEG5000‐(KKEEE)_3_K, (m) 90% PEG2000‐NT, or (n) PBS administration at 24 hours

In Figure 3c, we evaluated how the size of nanoparticles alters kidney targeting and compared micelles with varying PEG molecular weight.^50^ (KKEEE)_3_K micelles consisting of PEG1000 had renal accumulation of 2.43 × 10^9^ ± 2.0 × 10^8^ p/s/cm^2^/sr that was significantly greater than (KKEEE)_3_K micelles consisting of PEG5000 (1.28 × 10^9^ ± 2.3 × 10^8^ p/s/cm^2^/sr, *p* < .0001, as 45% PEG1000‐(KKEEE)_3_K micelles were found to be 10.4 ± 1.8 nm in diameter and near the reported renal filtration cut‐off size (Table 1, Figures 3c and 4). Similarly, PEG5000‐(KKEEE)_3_K showed lower renal accumulation compared to PEG20000‐(KKEEE)_3_K with 90% peptide density (1.28 × 10^9^ ± 2.3 × 10^8^ p/s/cm^2^/sr and 1.8 × 10^9^ ± 1.7 × 10^8^ p/s/cm^2^/sr, respectively, *p* < .0001). Hence, our results demonstrate a negative correlation between the size and renal targeting ability of PAMs.

The potential influence of the protein corona on micelles on GFB penetration will be further studied in the future to better understand the factors that affect nanoparticle kidney targeting.

### 2.3 Micelle colocalization with megalin

**FIGURE 5:**
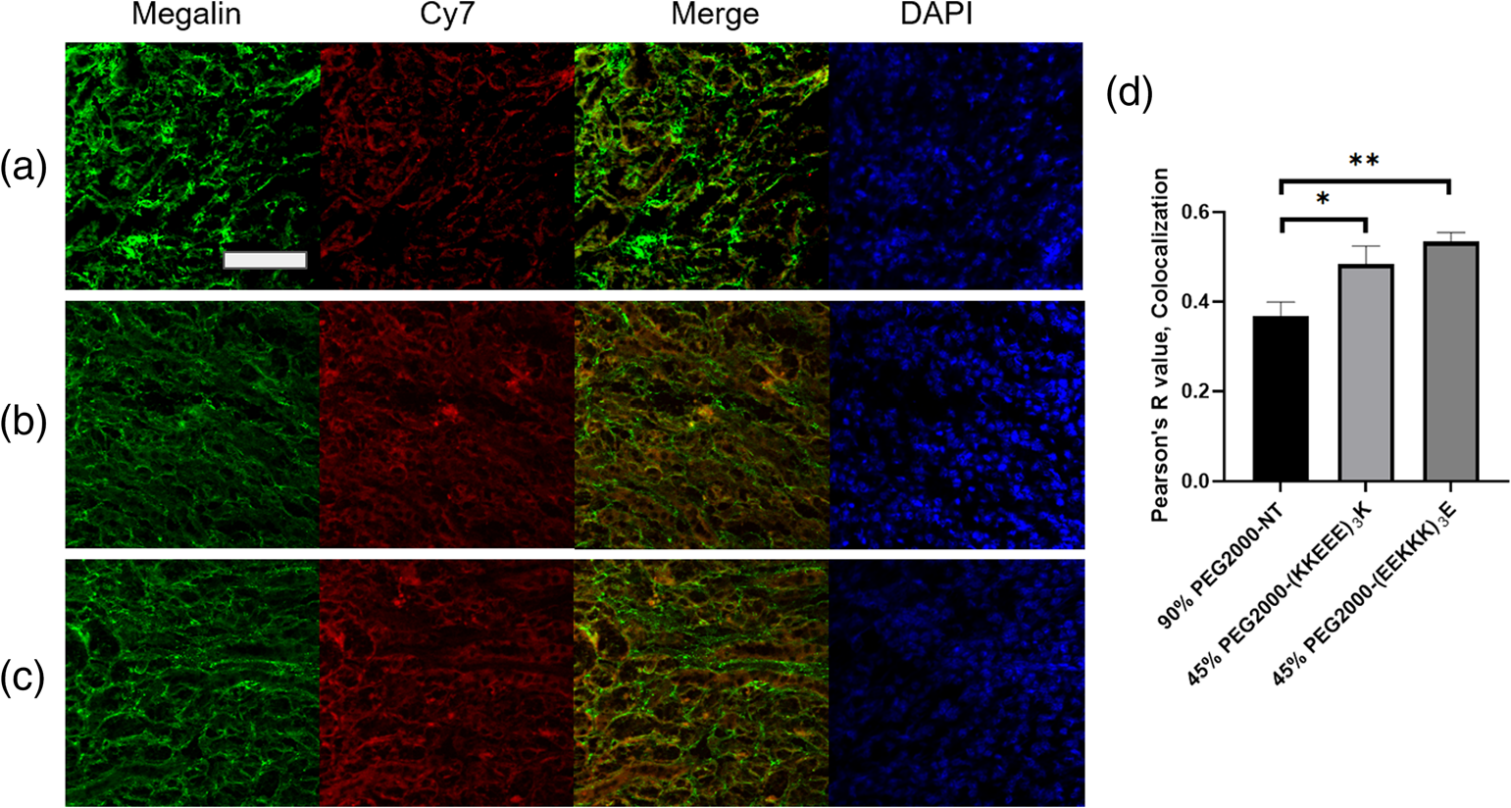
Megalin staining and colocalization with micelles in kidney section of mice administered (a) 90% PEG2000‐NT, (b) 45% PEG2000‐(KKEEE)_3_K, or (c) 45% PEG2000‐(EEKKK)_3_E. (d) Quantification of colocalization between micelles and megalin. Scale bar: 100 μm. *p < .05, **p < .01

As mentioned, previously, the (KKEEE)_3_K peptide was found to target the kidney in part through megalin, a multiligand receptor that is present on the plasma membrane of proximal tubule cells, and the (KKEEE)_3_K peptide showed significantly reduced uptake in megalin‐deficient mice.^19^, ^20^, ^51^, ^52^, ^53^ As demonstrated by Vegt et al, megalin has been reported to associate with a library of peptides (octreotide, octreotate, minigastrin, exendin, and neurotensin) of varying charges.^54^ To verify micelle binding to megalin, immunohistochemistry staining of kidneys of mice treated with 45% PEG2000‐(KKEEE)_3_K as well as 45% PEG2000‐(EEKKK)_3_E were assessed, and colocalization was calculated. A similar Pearson's *R* value, corresponding to colocalization, of 45% PEG2000‐(EEKKK)_3_E (0.53 ± 0.02) to megalin was shown with 45% PEG2000‐(KKEEE)_3_K (0.48 ± 0.04, not statistically significant) vs. the NT micelle (0.37 ± 0.03, *p* < .01, Figure 5), confirming that in addition to (KKEEE)_3_K PAMs, PAMs consisting of zwitterionic peptides of opposite charge also accumulate in the kidneys in part via megalin. This corresponds with the ex vivo results that showed 45% PEG2000‐(EEKKK)_3_E had similar kidney accumulation to 45% PEG2000‐(KKEEE)_3_K (Figure 3a).

### 2.4 Tissue morphology and kidney health

**FIGURE 6:**
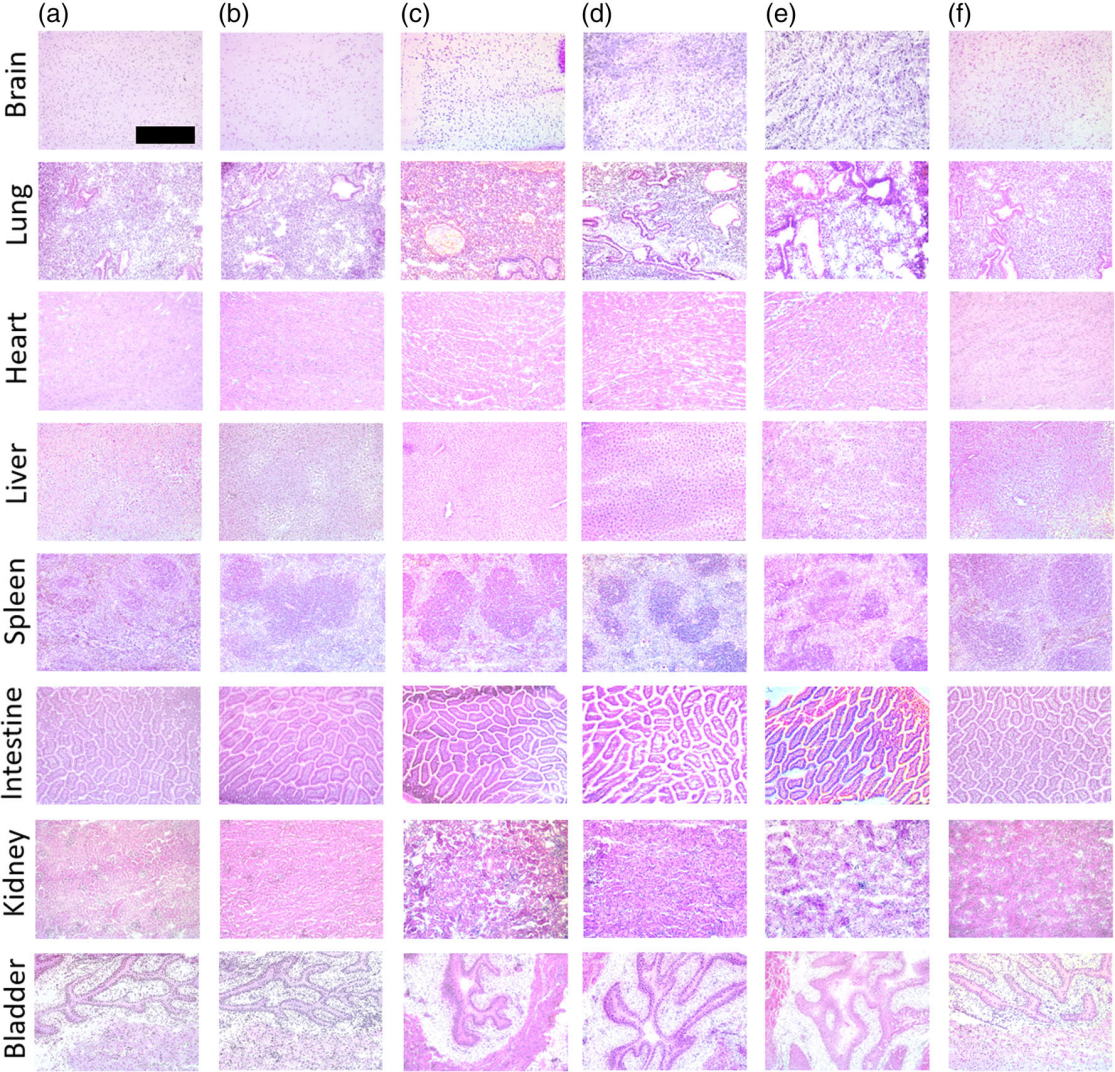
H&E staining of kidney sections 24 hr after (a) 45% PEG2000‐(KKEEE)_3_K, (b) 45% PEG2000‐(EEKKK)_3_E, (c) 45% PEG2000‐KKKKK, (d) 45% PEG2000‐EEEEE, (e) 90% PEG2000‐NT and (f) PBS administration. Scale bar: 100 μm

To assess the safety and biocompatibility of micelles and their eventual application as drug delivery vehicles, after ex vivo imaging, the brain, lung, heart, liver, spleen, intestine, kidney, and bladder were stained with H&E. In agreement with other PAM reports, the tissue morphology of all organs including the liver, intestine, and kidneys, where PAMs mostly accumulated, showed no tissue damage, and no significant difference was found to the PBS treatment group (Figure 6).^19^, ^55^, ^56^, ^57^, ^58^, ^59^

Kidney health and function of all the mice treated with micelles were further evaluated by assessing blood urea nitrogen (BUN) and urine creatinine levels. BUN levels for C57BL/6 mice with healthy kidney function is 25.0–75.0 mg/dl,^60^ and all the groups in this study were found to have BUN levels within this healthy range (Table 2). Similarly, the creatinine concentration in the urine indicates kidney health and urine creatinine levels for all groups were measured and found to fall within the healthy range for C57BL/6 mice (4.7 ± 3.1 mg/dl, Table 2).^61^

**TABLE 2:** BUN and urine creatinine levels upon micelle administration

NP	BUN (mg/dl)	Creatinine (mg/dl)
45% PEG2000‐(KKEEE)_3_K	40.8 ± 12.6	5.5 ± 1.8
45% PEG2000‐(KKEEE)_2_K	64.6 ± 33.3	5.7 ± 3.1
45% PEG2000‐(KKEEE)K	47.9 ± 13.9	4.2 ± 0.2
45% PEG2000‐(EEKKK)_3_E	35.2 ± 8.6	5.5 ± 1.9
45% PEG2000‐(EEKKK)_2_E	60.7 ± 30.4	9.0 ± 3.5
45% PEG2000‐(EEKKK)E	48.4 ± 12.8	4.5 ± 2.6
45% PEG2000‐KKKKK	41.4 ± 1.6	4.9 ± 0.4
45% PEG2000‐EEEEE	48.7 ± 4.3	5.6 ± 3.4
90% PEG2000‐(KKEEE)_3_K	29.5 ± 6.8	4.1 ± 1.7
90% PEG2000‐(EEKKK)_3_E	26.3 ± 16.0	3.9 ± 1.2
45% PEG**1000**‐(KKEEE)_3_K	39.5 ± 4.1	5.0 ± 3.1
90% PEG**5000**‐(KKEEE)_3_K	54.0 ± 10.9	5.1 ± 1.0
90% PEG2000‐NT	36.2 ± 5.5	4.4 ± 3.9
PBS	47.1 ± 9.4	7.6 ± 5.4

## 3 CONCLUSION

In this study, we altered the physicochemical properties of PAMs by developing a library of micelles containing various peptide sequences and molecular weight and assessed its effect on renal targeting. Micelles were first characterized by TEM and DLS before intravenous administration into wildtype mice. After 24 hours post‐administration, ex vivo imaging was conducted to compare kidney targeting ability. All PAMs tested in this study had high kidney accumulation, and our studies showed that nanoparticles with more zwitterionic amino acid repeats that are positively‐charged and close to the renal filtration cut‐off size tend to have higher renal accumulation. Furthermore, histological analyses confirmed no tissue damage, and kidney function levels of mice were within normal ranges. Overall, our studies indicate the potential of PAMs as targeting, nanocarriers for kidney applications and future work will focus on evaluating the mechanical properties and micelle concentration of nanoparticles on kidney targeting, as well as the influence of the protein corona on GFB penetration.^62^ Moreover, the therapeutic efficacy of drug‐loaded micelles and the contribution of nanoparticle delivery through the peritubular capillaries will be assessed in CKD mouse models.

## 4 MATERIALS AND METHODS

### 4.1 Micelle synthesis

Peptides were synthesized using standard Fmoc‐mediated solid phase peptide synthesis on an automatic PS3 peptide synthesizer (Protein Technologies, Tucson, AZ) with rink Amide resin (Protein Technologies, Tucson, AZ). A cysteine was added to the N‐terminus of all the peptide sequences in order to make a thioether linkage reaction. The peptides were then cleaved from the resin with 94:2.5:2.5:1 volume ratios of trifluoroacetic acid:1,2‐ethanedithiol:H_2_O:triisopropylsilane. Cleaved peptides were precipitated and washed several times with ice cold diethyl ether, dissolved in Milli‐Q water, lyophilized, and stored at −20°C. The crude peptides were purified by reverse‐phase high performance liquid chromatography (HPLC) (Shimadzu, Kyoto, Japan) on a C18 column (Phenomenex, Torrance, CA) at 55°C with 0.1% formic acid in acetonitrile/water mixture. The purified peptides were characterized using matrix‐assisted laser desorption ionization time‐of‐flight mass spectral analysis (MALDI‐TOF) (Autoflex speed, Bruker, Billerica, MA) and conjugated to 1,2 distearoyl‐sn‐glycero‐3‐phosphoethanolamineN‐[maleimide(polyethylene glycol)‐1000/2000/5000], or DSPE‐PEG(1000)‐maleimide/DSPE‐PEG(2000)‐maleimide/DSPE‐PEG(5000)‐maleimide (Avanti Polar Lipids, Alabaster, AL) via a thioether linkage by mixing an equimolar amount of the lipid and pure peptide in Milli‐Q water (pH 7.2) at room temperature for over 24 hours with gentle agitation. The mixture was further purified by a C4 column (Phenomenex, Torrance, CA) and characterized by MALDI‐TOF as described above. The fluorophore‐conjugated monomer was synthesized by mixing an equimolar amount of Cyanine7 NHS ester (Lumiprobe, Hunt Vally, MD) with 1,2‐distearoyl‐sn‐glycero‐3‐phosphoethanolamineN‐[amino(polyethylene glycol)‐1000/2000/5000] (ammonium salt) or DSPE‐PEG(1000)‐amine/DSPE‐PEG(2000)‐amine/ DSPE‐PEG(5000)‐amine (Avanti Polar Lipids, Alabaster, AL) in 0.1 M sodium bicarbonate solution (pH 8.3) at room temperature overnight. The mixture was purified on a C4 column and characterized by MALDI‐TOF as described above.

13 types of micelles were synthesized via self‐assembly. Micelles consisting of varying molar ratios of amphiphiles were synthesized: 100% DSPE‐PEG‐peptide, 50:50 molar ratio of DSPE‐PEG‐methoxy:DSPE‐PEG‐peptide, 90:10 molar ratio of DSPE‐PEG‐peptide:DSPE‐PEG‐Cy7, and 45:45:10 molar ratio of DSPE‐PEG‐methoxy:DSPE‐PEG‐peptide:DSPE‐PEG‐Cy7. All monomers were dissolved in methanol or chloroform and evaporated with nitrogen to form thin films. Thin films were dried overnight under vacuum and hydrated at 80°C for 30 min. Micelles prepared for DLS, zeta potential measurements, and TEM were hydrated in Milli‐Q water, whereas micelles prepared for in vivo administration were hydrated in phosphate buffered saline (PBS).

### 4.2 Dynamic light scattering (DLS) and zeta potential measurements

Micelles containing 100% DSPE‐PEG‐peptide or 50:50 by molar ratio DSPE‐PEG‐methoxy: DSPE‐PEG‐peptide were synthesized and assessed via DLS and zeta potential measurements. Micelle diameter and zeta potential was measured using Zetasizer Ultra (Malvern Instruments, Malvern, UK) at a micelle concentration of 100 μM in Milli‐Q water (*n* = 4) immediately after micelles were hydrated from thin films.

### 4.3 Transmission electron microscopy

Seven microliters of 100 μM micelles solution in Milli‐Q water was placed onto a 200‐mesh carbon TEM grid (Ted Pella, Redding, CA) for 5 min. Then, the grid was washed with Milli‐Q water and negatively stained with 2 wt.% uranyl acetate solution (Polysciences, Warrington, PA). The staining solution was wicked away after 2 min and the grid was washed with Milli‐Q water again. The grid was dried overnight under room temperature in the dark and imaged on a JEOL 2100F (JEOL, Tokyo, Japan).

### 4.4 In vivo renal targeting and biodistribution

To test the renal targeting ability in vivo, 100 μl of 1,000 μM micelles containing 90:10 molar ratio of DSPE‐PEG‐peptide:DSPE‐PEG‐Cy7 or 45:45:10 molar ratio of DSPE‐PEG‐methoxy:DSPE‐PEG‐peptide:DSPE‐PEG‐Cy7 or PBS were tail vein injected with in 6–7 week old male and female C57BL/6 mice (*n* = 4, Jackson Laboratories, Bar Harbor, ME). After 24 hours in circulation, mice were euthanized and their organs (i.e., brain, heart, lungs, liver, spleen, intestines, kidneys, and bladder) were harvested and imaged ex vivo via Ami HTX (Spectral Instruments Imaging, Tucson, AZ). Qualification of the fluorescence signal was conducted to determine the biodistribution of particles by Aura imaging software (Spectral Instruments Imaging, Tucson, AZ). All animal experiments were approved by University of Southern California (USC) Institutional Animal Care and Use Committee (IACUC).

### 4.5 Histology

Immediately after ex vivo imaging, harvested organs were flash frozen in 2‐methylbutane and liquid nitrogen, embedded in optimum cutting temperature (OCT) compound, and sectioned into 8 μm samples via a CM3050 S Cryostat (Leica CM3050S, Leica, Wetzlar Germany). Tissue sections were then stained with hematoxylin and eosin (H&E) and imaged with a microscope (Leica DMi8, Leica, Wetzlar, Germany).

### 4.6 Immunohistochemistry

To assess micelle colocalization with megalin, kidney tissue sections were first washed with Tris buffered saline (TBS) plus 0.025% Triton X‐100 with gentle agitation for 10 minutes. A block buffer with 1% bovine serum albumin (BSA), 10% normal goat serum, and 0.3 m glycine in 0.1% PBS Tween was applied to the slides for 1 hour at room temperature. Then, the slides were applied with an anti‐Lrp2/Megalin antibody (Abcam, Cambridge, UK, 1:100) overnight at 4°C. The following day, slides were rinsed twice with TBS plus 0.025% Triton for 5 minutes and applied with fluorophore‐conjugated secondary antibody‐goat, anti‐mouse IgG H&L Alexa Fluor® 488 (Abcam, Cambridge, UK, 1:1000) for 1 hour at room temperature in the dark. Slides were then counterstained with DAPI and mounted with VectaMount™ mounting medium (Vector Laboratories, Burlingame, CA). Fixed slides were imaged with a LSM 700 confocal microscope (Zeiss, Oberkochen, Germany), and the colocalization between Cy7 and Alexa Fluor® 488 channels was performed on ImageJ with coloc2.

### 4.7 Kidney health

Renal health was assessed by analyzing blood urea nitrogen and urine creatinine levels in serum and urine. BUN was analyzed by a BUN enzymatic kit (Bioo scientific, Austin, TX), and urine creatinine was assessed by a mouse creatinine enzymatic kit (Crystal Chem, Elk Grove Village, IL).

### 4.8 Statistical analysis

All statistical analyses were performed using GraphPad Prism 8 (San Diego, CA). Analysis of variance (ANOVA) with a Tukey's test for post‐hoc analysis was used to determine statistical significance and *p* ≤ .05 was considered to be significant.

## CONFLICT OF INTEREST

The authors declare no potential conflict of interest.

## Supporting information


**Appendix** **S1**: Supporting information.
