# Supplementary material for: The effect of size, charge, and peptide ligand length on kidney targeting by small, organic nanoparticles
Source: Bioeng Transl Med. 2020 Jul 30;5(3):e10173. doi: 10.1002/btm2.10173 (PMC7510478; doi:10.1002/btm2.10173)
Supplement: Supplementary file 1 — Appendix S1: Supporting information. [file BTM2-5-e10173-s001.docx]

**Supporting Information**

**Size and Charge Effects of Small, Organic Nanoparticles on Kidney Targeting**

Yi Huang^1^, Kairui Jiang^1^, Xuting Zhang^1^, Eun Ji Chung*^123456^

^1^Department of Biomedical Engineering, University of Southern California, Los Angeles, CA, USA

^2^Department of Chemical Engineering and Materials Science, University of Southern California, Los Angeles, CA, USA

^3^Department of Medicine, Division of Nephrology and Hypertension, University of Southern California, Los Angeles, CA, USA

^4^Department of Surgery, Division of Vascular Surgery and Endovascular Repair, University of Southern California, Los Angeles, CA, USA

*Corresponding author

Department of Biomedical Engineering

University of Southern California

1002 Childs Way, MCB 357

Los Angeles, CA, 90089, USA

email: eunchung@usc.edu


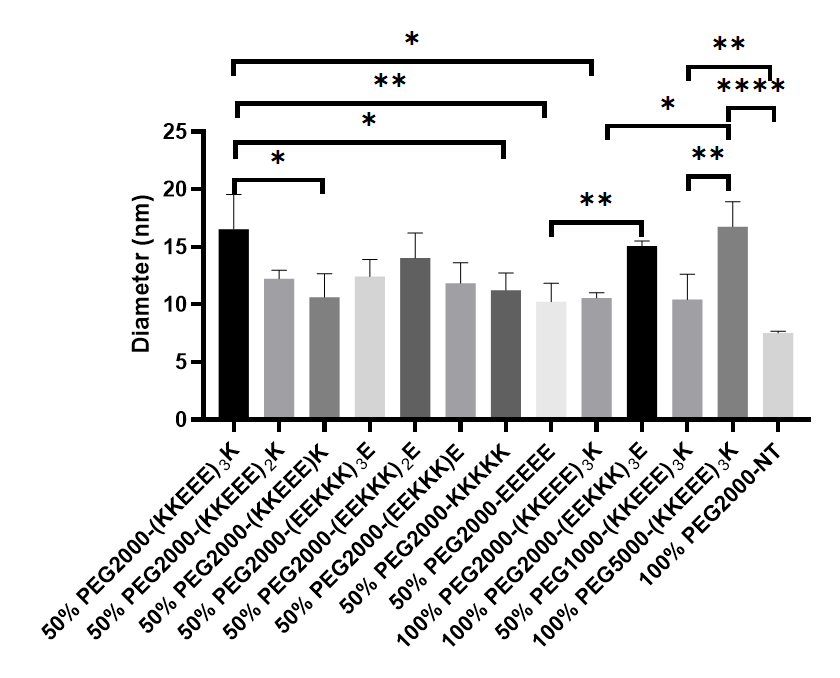


Figure S1. Comparison of hydrodynamic diameters of all micelles (* *p* < 0.05, ** *p* < 0.01, **** *p* < 0.0001).


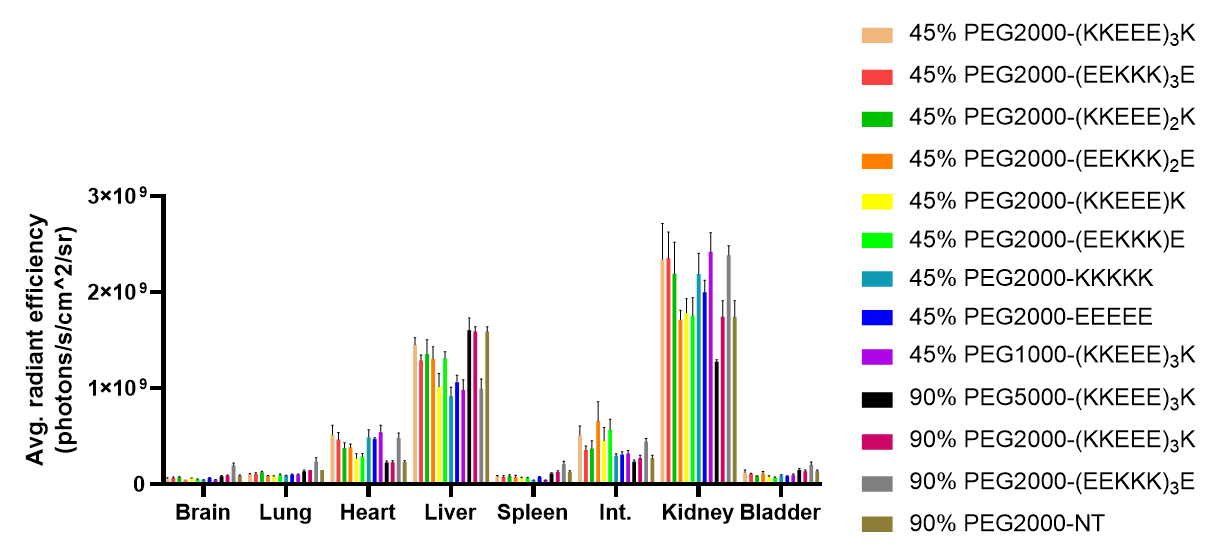


Figure S2. Biodistribution of all the micelles 24 hours after intravenous administration.


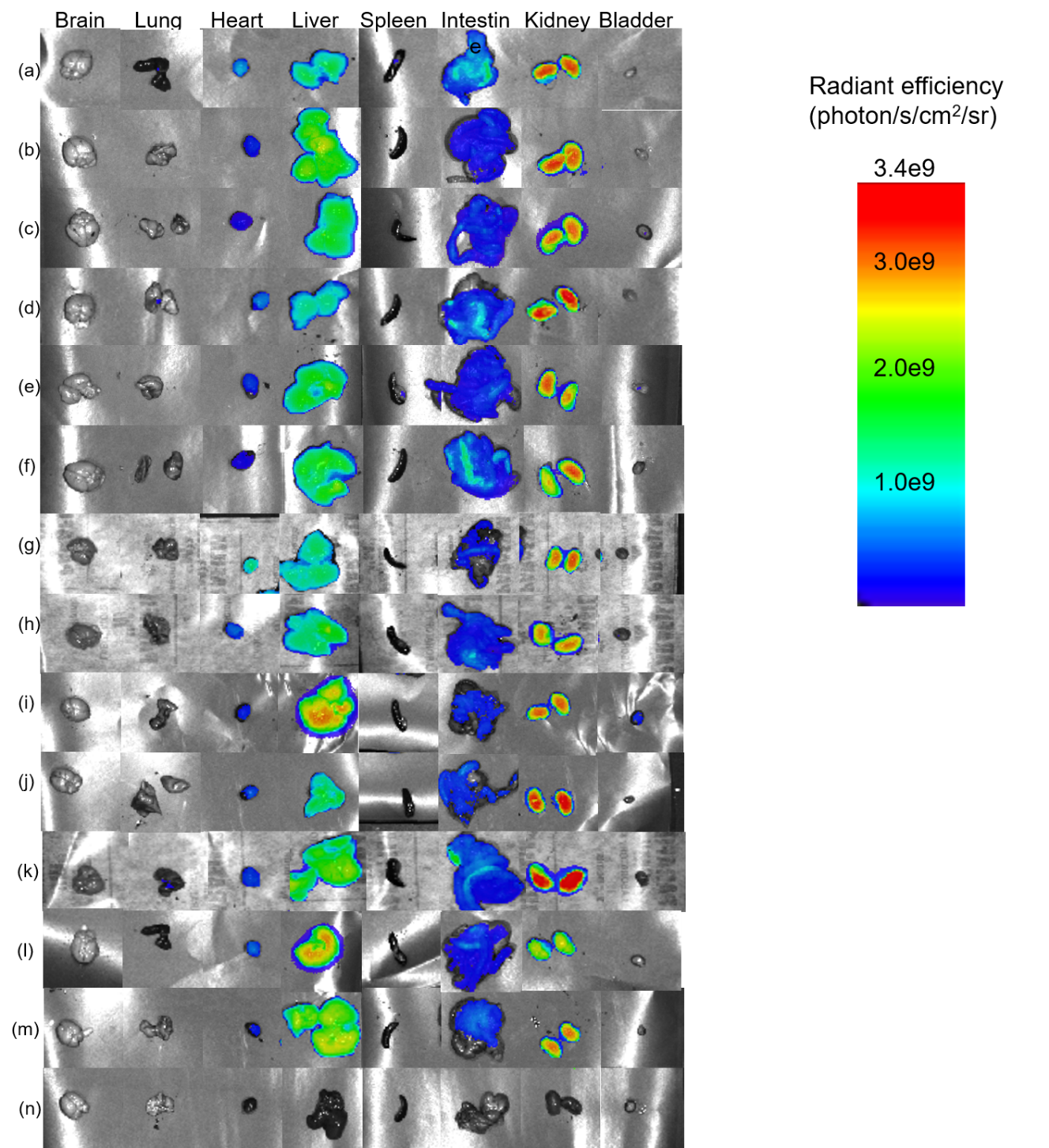


Figure S3. Ex vivo biodistribution of mice treated with (a) 45% PEG2000-(KKEEE)_3_K, (b) 45% PEG2000-(KKEEE)_2_K, (c) 45% PEG2000-(KKEEE)K, (d) 45% PEG2000-(EEKKK)_3_E, (e) 45% PEG2000-(EEKKK)_2_E, (f) 45% PEG2000-(EEKKK)E, (g) 45% PEG2000-KKKKK, (h) 45% PEG2000-EEEEE, (i) 90% PEG2000-(KKEEE)_3_K, (j) 90% PEG2000-(EEKKK)_3_E, (k) 45% PEG1000-(KKEEE)_3_K, (l) 90% PEG5000-(KKEEE)_3_K, (m) 90% PEG2000-NT, or (n) PBS.


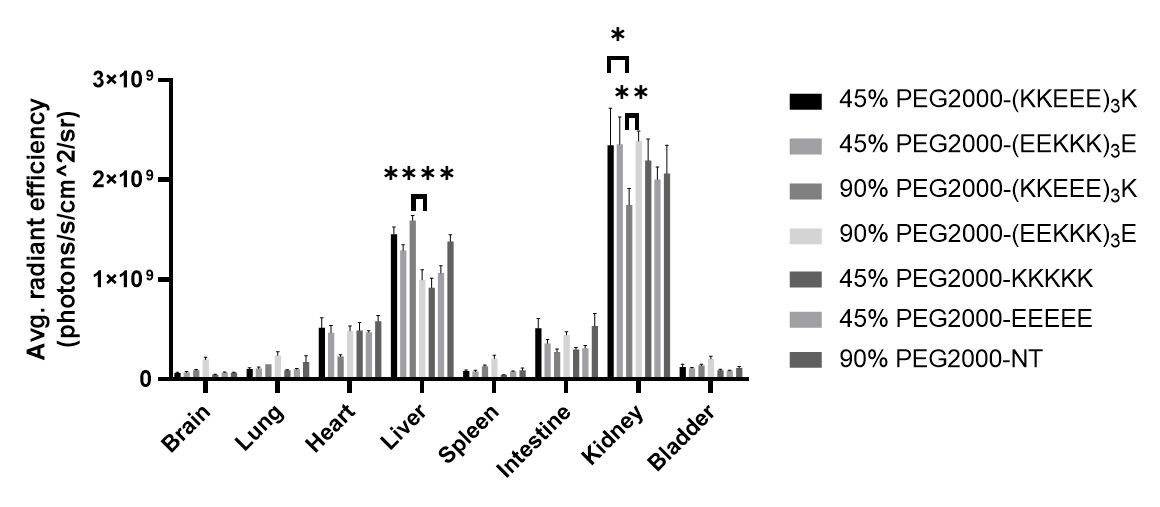


Figure S4. Biodistribution of micelles containing charge differences (* *p* < 0.05, ** *p* < 0.01, **** *p* < 0.0001).
